# Cases of Treatment of Disease by the Use of the Saratoga Water

**Published:** 1840-05-11

**Authors:** Milo L. North

**Affiliations:** Saratoga Springs


### *Cases of treatment of Disease by the use of the Saratoga Water.* By Milo L. North, M. D., of Saratoga Springs.

*Saratoga Springs, 8th May*, 1840.

To the Editors of the Medical Examiner.

Gentlemen:—I forward you a few extracts from my medical case book, kept here in the season of 1838 and 1839. The language, especially of the symptoms, is very nearly as it was taken during the hurry and pressure of our short and busy season.

Respectfully yours, Milo L. North.

Case I.—*Dyspepsia, with Emaciation and Night-Sweats.*

*August 17th*, 1839.—C. A. B., a merchant, from western New York, aged about forty-two. Has dry, short cough; great debility; sweats towards morning; bowels irregular; secretion of urine variable; pulse eighty-four, and soft; tongue foul; appetite indifferent; is losing flesh.

*Treatment.*—℞. Pulv. Antimon:

Gum. Acaciæ,

Sacch. Albi., āā ʒi.

Misce. ft. pulv., and divide in chartulas 12. Take one, followed by simple water, before each meal, and at bedtime. Two teaspoonfuls Epsom salts, and six tumblers from the iodine spring every morning, early. A hot-bath daily, of fifteen minutes, at 110°.

*August 21st.*—Pulse seventy-two, and soft. Tongue improved surprisingly. Has taken ten tumblers from the iodine spring, mornings, in addition to his salts, without the least oppression or colic, and with free evacuations.

*August 24th.*—Pulse sixty-six, and easy. Cough much diminished, and loose. Free bowels. Fur on tongue nearly gone. The hot bath agreeable; is uniformly followed by free perspiration, several hours. Still finds ten tumblers agreeable, and thoroughly cathartic and diuretic. The secretion of urine, regular and abundant. Has no need of the sulphate of magnesia. Appetite strong.

Mr. B. gained so rapidly, that he soon left for home. On his arrival there he soon began to lose ground, although he had a full supply of iodine water in bottles, and took it liberally. It soon occurred to him that he was indebted chiefly to the hot baths for his rapid amendment here. He wrote me that he recommenced the use of the hot water in a rude tub soon after; and his improvement was so unequivocal that he was about constructing a regular bath,—and if that did not produce the wished-for effect, he should immediately return to Saratoga. As I have heard nothing from him since, I conclude the bath, which is often ranked as an auxiliary, and often an ambiguous remedy, became to him the primary and efficient means of his recovery.

Case II.—*Scrofulous Ulceration in various parts of the body, accompanied with Exfoliation of the Bones.*

*August 23d*, 1839.—J. S. K., from Boston, aged seventeen. Four years since, had what was called typhus fever. Since then has had ulcers in various parts of the body. Has had an ulcer on the upper part of the thigh steadily for three years. The opening fistulous, with a suppurating cavity of several inches around the fistula. The pus thick and yellow. Had hip disease five weeks last spring. Treated by Dr. Haywood. Could not touch his foot to the ground. At one time had stiff neck four months. Pieces of bone have come out from various places. Appetite poor. Pulse eighty-six, and soft. Tongue foul. *Treatment.*—The iodine water taken every morning at the spring, sufficient to prove mildly laxative. Also, one tumbler from the Flat Rock chalybeate spring, at 12 M., and at 5 and 9 P. M. In addition to these, a bath of mineral water every second day, for fifteen minutes, at 110°.

*August 27th.*—Pulse ninety-two, and soft. Tongue clean. Baths agreeable after the first three or four minutes.

*September 17th.*—Can walk two and a half miles as easily as half a mile when he arrived. Has much more vivacity, as well as strength. Increasing in flesh. Feels every symptom of convalescence. On his own responsibility, guided by his own perceptions of the efficacy of the baths, he increased the temperature to 112°. Continues the iodine and Flat Rock waters. Expects soon to return home, with a full determination to spend the ensuing summer in Saratoga.

Case III.—*Inflamed Prostate.*

*September 14th*, 1839.—Gen. R., from Connecticut, about fifty or fifty-five. Had a severe and dangerous inflammation of the prostate gland last March, which caused total retention of urine. At the end of five days, after many trials with the catheter and bougie had failed, five quarts (!) of urine were drawn off by a catheter of peculiar construction. Since that time Gen. R. has been feeble and very bilious. No return of the constitutional powers, although the obstruction is not now troublesome. Pulse seventy-eight, and hard. Face sallow. Vomits bile. *Treatment.*—Two blue pills at bedtime for three nights: afterwards, one a night. Six to eight tumblers Congress water in the morning. Bath every second day from 90° to 96°.

*Sept. 25th.*—Pulse rather wiry. Some pain in the shoulder. Sallowness of countenance wholly removed. Eats and sleeps better.

℞.—Sub. Mur. Hyd., gr. 1½.

Opii, gr. ¼.

Ft. pilula, sumenda hora somni.

One piling teaspoonful of calcined magnesia, and three tumblers Congress water in the morning. Omit blue pill.

The patient left a few days after this date, very much improved. When he arrived, had the assistance of his wife's arm in ascending stairs; now walks vigorously, alone, up hills, and at considerable distances. As the new movements produced in the animal economy by these waters often continue many months after the invalid leaves us, it is all but certain that this gentleman recovered his usual health in the course of the autumn.

Case IV.—*Chronic Colitis.*

*September 11th*, 1839.—Mr. J. L. C., of Georgia, say thirty-five years of age. Has had fixed pain in the right side for years, passing up to the right shoulder. This pain was preceded many years by chronic diarrhœa. Tongue furred. Great variety of alvine evacuations. Has constant uneasiness around him in the region and direction of the diaphragm. Pulse fifty-six, and easy. Has passed five summers at the Virginia Springs. Pain at epigastrium, in the region of the great arch of colon, not relieved at the Springs, but thinks he passes the following winter better for his visit in the summer. *Treatment.*—An opium and calomel pill, night and morning. A hot bath every evening at 8, going thence to bed. A bottle of Congress water in the room while dressing, and three tumblers at the spring, all before breakfast.

*Sept. 13th.*—Bath agreeable, at 100°. Thorough cathartics. Some griping.

*Sept. 17th.*—Has so little pain now, that without my direction, omitted calomel and opium pills. Felt languid and feeble while in the bath, but this was followed by a fine glow and very comfortable sensations for many hours. Appetite decidedly better. Pain of colon "almost forgotten."

*Sept. 19th.*—Although no opium since 15th, pain scarcely felt. Is entirely free from it most of the time. A decidedly improved feeling through the abdomen, every way. Operation of the water very easy and generous. The baths highly agreeable, and he determines to construct a bath at home. Skin more natural.

*Sept. 21st.*—Bath yesterday at 102°, very grateful. Still improving in every respect. Left soon after, on account of the lateness of season and the approach of business, resolving to spend the whole of next summer here if his health should not be fully restored by the restorative effects of the waters already used.

Case V.—*Enlarged Tonsils.*

This case being that of a boy from one of our western cities, is detailed in a small book just published, entitled "Saratoga Waters;" and is alluded to here to call the attention of the faculty to those instances of this affection where excision of the tonsils fails through general derangement of the system. This boy was left, for the summer, under my care, and fully recovered, going home with a new constitution. He lost no time in his education, being placed in one of our academical schools. The water was taken as an alterative, combined with a daily shower bath.

Case VI.—*Chronic Affection of the Brain and Spinal Marrow.*

*July 26th*, 1839.—Mrs. P. C., from Connecticut, aged about thirty-seven. Has at times lost her reason. Has continual *cephalalgia.* Her whole arms feel permanently as others' feel when they have "hit their elbow." Numbness of lower extremities, and great loss of muscular power. Amenorrhœa. Pulse good. Wakefulness. Indigestion. She was directed to take ʒi. antimonial wine at bedtime, and from six to eight tumblers Congress water in the morning.

*Aug. 13th.*—The use of the water has produced a wiry pulse, and great pressure of the head, and stricture of the lungs. After bleeding her 16 oz., I put her on an active course of purgatives, antimonials, extract of hyosciamus, &c., suspending the Congress water, and, in two or three weeks, she was rapidly convalescing, and left here with such health as she had not known for years. Although the Congress water proved too tonic, it may have effected a condition of the fluids and solids that enabled the common remedies to have much more power than before her trial of the waters.

Case VII.—*Chronic Affection of the Brain and Spinal Marrow.*

*August 1st*, 1839.—Miss J. S., a single lady, aged twenty-seven, an adopted daughter of a gentleman in one of the cities of this state. Has been an invalid since the age of 17. For the last seven years nearly helpless, and for the last four years has constantly had a special nurse. If she rides out, she is carried to and from the chaise by her adopted father. She spends the day in a cradle made expressly for her. If she journies, this must form part of her travelling apparatus; and in this cradle I always found her when I paid my visits to her in this village. She converses in a whisper. The optic nerves are so exquisitely painful, that she lies much of the time with her fingers pressing on her eyeballs. The nerves generally are neuralgic. All her senses morbidly acute. So is the memory. She recollects, verbatim, the letters she wrote five years ago. Pulse seventy-four, soft, equable. Nothing morbid in tongue or countenance. Digestion, a cypher. Catamenia failed a year since. Her nights very tedious. Has tried many remedies and expedients suggested by the untiring kindness of her friends, and patience of her physicians.

To remove this long-continued and formidable disease, two things were attempted: 1st, to institute a revulsion from the cerebro-spinal system; and, 2d, to administer the mineral waters as an alterative and tonic during the action of these revulsive remedies. The first indication was fulfilled by, 1st, a hot bath, every second day, of 110°, made of the water from the saline fountains, and continued fifteen minutes. The operation of these baths was unequivocally pleasurable and refreshing at the time, with the exception of some languor during the first hour. As they were followed by several hours' free perspiration, and as several pounds of extra blood were confined in the cutaneous vessels during the same time, it will be very apparent that these hot baths must have formed a powerful counteraction to the disease of the nervous apparatus. They appeared, moreover, decidedly beneficial to the patient at the time. 2d. An efficient and continued perturbation of the stomach, liver, and bowels, by daily doses, in the morning, of magnesia, rhubarb, and some carminative. 3d. Small doses of opium and calomel, to diminish the extreme susceptibility of the nerves and her general neuralgic sufferings; to produce a new action in the liver, and augmentation of bile; and, also, to cause direct revulsion from the brain by a temporary, factitious inflammation of the membranes of the mouth by ptyalism. 4th. Several doses daily of camphorated tincture of opium and antimonial wine, to produce perturbation in the stomach, liver, and upper parts of the alimentary canal.

While these multiplied impressions were being made on the whole muco-cutaneous surface, and upon the chylopoietic viscera, the second indication was fulfilled by her taking, before each meal and at bedtime, one or two tumblers of water, fresh from the Congress spring, as an alterative and tonic. This quantity probably lingered longer in the circulating mass, and effected greater changes in it, than three times the quantity would have done.

By the 15th of August, it was evident the patient had imbibed strong hopes of recovery. Every symptom was improved. Her *sensations* were new, and full of evidence of a favourable modification of all the processes of the system. After staying a few days longer, she returned home; and the progress of her recovery to a late date, is detailed in the following letter from her uncle and adopted father to myself:

March 10, 1840.

*Dr. North*—Dear Sir: Without any apology for my long delay, I will proceed directly to my object of informing you of the health of your patient of last summer, Miss J. S. You will recollect her and her cradle at Wilcox's. Soon after her return, she repeated the salivating course she had pursued under your prescription in Saratoga, though I believe you thought the repetition unnecessary. On the whole, she has been improving from that time to this. She has taken her hot bath almost every day since she left you; and, for four months past, has taken nearly the same kind of food with the rest of the family. Tea, fat pork, and sometimes beef in small quantities, potatoes, bread and butter, are among her articles of diet. Exercise in the open air almost daily, not excepting the coldest days of winter, by riding in a covered sleigh, and late last fall sometimes on horseback, has been rigidly enforced. She has performed some short journeys. Her lungs and chest are in such a state, that she can and does talk a good deal. She dismissed her cradle late in the fall. She sits up sometimes several hours together. Her common practice is to spend the day in alternately sitting up an hour or two, and then lying down.

To-day she and I had quite a game at battledoor, although this was not the first time of *her* performing it. She was able to continue it a few minutes with great sprightliness. I forgot to mention that at one time last fall she rode seven miles on horseback. I have great hope she will ultimately recover comfortable health. * * * She thinks the *hot* bath has been particularly efficacious to her relief.

Respectfully yours, H. C.

